# Ovarian hyperstimulation syndrome: a clinical report on 4894 consecutive ART treatment cycles

**DOI:** 10.1186/s12958-015-0067-3

**Published:** 2015-06-23

**Authors:** Mário Sousa, Mariana Cunha, José Teixeira da Silva, Cristiano Oliveira, Joaquina Silva, Paulo Viana, Alberto Barros

**Affiliations:** Department of Microscopy, Laboratory of Cell Biology, Institute of Biomedical Sciences Abel Salazar, University of Porto (ICBAS-UP), Rua Jorge Viterbo Ferreira, 228, 4050-313 Porto, Portugal; Multidisciplinary Unit for Biomedical Research-UMIB, ICBAS-UP, Rua Jorge Viterbo Ferreira, 228, 4050-313 Porto, Portugal; Centre for Reproductive Genetics Alberto Barros, Av. do Bessa, 240, 1° Dto. Frente, 4100-012 Porto, Portugal; Department of Genetics, Faculty of Medicine, University of Porto (FMUP), Alameda Prof. Hernâni Monteiro, 4200-319 Porto, Portugal; Institute of Health Research an Innovation, University of Porto, Porto, Portugal

**Keywords:** Gonadotrophin-releasing hormone agonist, ICSI, IVF, Ovarian hyperstimulation syndrome, Embryological, Clinical and newborn outcomes

## Abstract

**Background:**

Although a large number of studies have been dedicated to ovarian hyperstimulation syndrome (OHSS) none gave full embryological and clinical outcomes comparing oocyte trigger with human chorionic gonadotrophin (HCG) versus with a gonadotrophin-releasing hormone (GnRH) agonist (Buserelin) in cases with suspicious OHSS. The aim of the present study was thus to analyze 4894 consecutive assisted reproductive treatment cycles to undercover associated risk factors for development of OHSS, and the effects of the use of Buserelin as ovulation trigger on embryological and clinical outcomes.

**Methods:**

In the 51 cases that developed OHSS, ovulation trigger was performed with HCG as indicators were not suspicious for OHSS. These were compared against two types of groups: 71 cases where Buserelin was used for ovulation induction due to suspicious development of OHSS; and those remaining 4772 cases where ovulation trigger was currently performed with HCG (control).

**Results:**

Of the cases treated with Buserelin the oocyte maturation rate and the ongoing pregnancy rate were significantly lower, with higher rates of ectopic pregnancy and newborn malformations, but none developed OHSS. Of the OHSS cases, 23 needed hospitalization, with no major complications.

**Conclusions:**

Young age, lower time of infertility, lower basal follicle stimulating hormone levels, higher number of cases with female factor and polycystic ovarian syndrome, high number of follicles and higher estradiol concentrations were the risk factors found associated with OHSS. Cases with OHSS also presented higher follicle count but the estradiol levels were within the normal range. It thus remains to develop more strict criteria to avoid all cases with OHSS.

## Background

Ovarian hyperstimulation syndrome is an excessive response to controlled ovarian hyperstimulation during treatment cycles by assisted reproduction technologies (ART), either using in-vitro fertilization (IVF) or intracytoplasmic sperm injection (ICSI), being the most important iatrogenic complication of the treatment stimulation protocol [1, 2].

During ART treatments, controlled ovarian stimulation leads to multiple oocytes and supra-physiological levels of estrogens. The presence of OHSS is characterized by enlargement of the ovaries associated with ascites due to an increased peritoneal capillary permeability [3]. It presents a very broad spectrum of clinical manifestations, with a incidence of 0.2-1 % of all stimulation cycles, from mild to moderate symptoms (about 3-6 % of the ART treatment cycles), which only requires vigilance [2], to more severe symptoms (about 1 % of the treatment cycles) that often requires hospitalization [4].

Severe OHSS may be associated with hepatic and respiratory failure [2], and with an increased risk of venous thromboembolism that can be fatal [5–9]. Due to these severe outcomes, the recognition of risk factors for the development of OHSS became crucial. These have been postulated to rely on young age, low body mass index (BMI), presence of polycystic ovarian syndrome (PCOS), high basal anti-Mullerian hormone (AMH) concentrations, high number of antral follicles, development of multiple follicles, number of collected oocytes, high serum estradiol (E2) levels, blood group A, mutations in bone morphogenic protein (BMP) alleles, presence of follicle stimulating hormone (FSH) receptor mutations and polymorphisms, high FSH dosage, high ratio between luteinizing hormone (LH)/FSH, high human chorionic gonadotrophin (HCG) dosage, high inhibin A and inhibin B values, high vascular endothelial growth factor (VEGF) and other interleukins levels, decreased alpha-2-macroglobulin (inhibitor of VEGF) levels, and allergies [1–4, 10–12]. Presently, the main risk factors used to precociously identify the risk of developing OHSS are the presence of PCOS, the number of developed follicles, and the rising levels of E2 concentrations. The use of a GnRH agonist as ovulation trigger has been advocated to avoid OHSS completely [13–15]. Nevertheless, OHSS can develop at a later stage, as there are cases that evade diagnosis. A personalization of the stimulation protocols are thus of the utmost importance [12].

In the present work we retrospectively analyzed 4894 ART treatment cycles to evaluate the risks for development of OHSS. For this, patient demographic and stimulation features, embryological and clinical data were evaluated. The use of a GNRH agonist as ovulation trigger was also evaluated to analyze its effects on embryological and clinical outcomes.

## Methods

In accordance to the National law on Medical Assisted Procreation (Law n.° 32/2006, 26 July) and the requisites of the National Council on Medical Assisted Procreation (CNPMA, 2008), data bases were used after patient informed and written consent.

### Participants

We performed a retrospective analysis of 4894 ART consecutive treatment cycles (from 2005 to 2011). Cases that developed OHSS in which ovulation trigger was performed with HCG as the monitoring indicators (serum levels of E2 ≤ 4000 pg/ml; ≤ 20 follicles) were not suspicious for the development of OHSS, were compared against two types of groups: those where a GnRH agonist (Buserelin) was used for ovulation induction due to suspicious development of OHSS; and those remaining cycles where ovulation trigger was currently performed with HCG (control). In all OHSS cases development of symptoms and signs only occurred after pregnancy confirmation (late OHSS). Also, in all cases, none of the patients in the three groups had previous OHSS, which helped to exclude this as a potential risk factor as previously reported [2, 4, 16]. Data on patient demographic and stimulation characteristics, and embryological and clinical outcomes were evaluated. No significant differences were found regarding patient karyotypes.

### OHSS classification

*Mild OHSS* is characterized by the presence of increased weight, thirst, abdominal discomfort and/or distension, with an ovary volume of < 5 cm. *Moderate OHSS* is characterized by the additional presence of nausea and vomiting, painful abdominal distension, dyspnea and/or ascites (detectable by ultrasound), with an ovary volume of 5–12 cm, without need for hospitalization. *Severe OHSS* is characterized by the presence of vomiting (sometimes uncontrollable), dyspnea, accumulation of fluid in the third space with hydrothorax and/or tense ascites (with pain) with evidence of intravascular fluid loss, hypovolemia with hemoconcentration, electrolyte imbalance, oliguria and/or hepatorenal failure, with an ovary diameter > 12, with need for hospitalization [17, 18].

### Stimulation protocol

Women underwent controlled ovarian hyperstimulation with a GnRH antagonist (cetrorelix; Merck Serono, Geneve, Switzerland; ganirelix; Organon, Oss, Netherlands) short protocol. For stimulation, recombinant FSH (rFSH) was used (Puregon; Organon; Gonal-F; Merck Serono). About 36 h before oocyte recovery HCG (5000-10,000 IU, im/sc; Pregnyl; Organon) or Buserelin (0.8 cc; Suprefact; Sanofi Aventis, Frankfurt, Germany) were administered. Estradiol serum levels were assayed at the day or one day before HCG or GnRH agonist administration [19, 20].

### Gamete and embryo handling

Gamete and embryo handling was performed with media from Medicult (Jyllinge, Denmark) or Vitrolife (Kungsbacka, Sweden). Microinjection was performed in an inverted microscope (Nikon DIAPHOT 200; Nikon, Tokyo, Japan), equipped with a thermal plate (37 °C), Hoffman optics (Nikon) and Narishige micromanipulators (MO-188; Narishige, Tokyo, Japan), using micropipettes from Swemed (Goteborg, Sweden). ICSI was performed using the strong dislocation of the cytoplasm [21]. Embryo grade was evaluated according to described methods [22, 23]. Embryo transfer was performed under ultrasonography, using a Sure View Wallace Embryo Replacement Catheter or Wallace malleable stylet (Smiths Medical Int, Kent, UK).

### Luteal supplementation

All patients had luteal supplementation with intravaginal administration of 200 mg of natural-micronized progesterone, 8/8 h (600 mg day) (Jaba, Besins Int, Montrouge, France) beginning on the day of oocyte retrieval. Implantation was confirmed by a rise in serum βHCG 12 days after embryo transfer. Progesterone was maintained until βHCG serum assay and, if positive, it was continued until 12 weeks of gestation. Clinical pregnancy was established by ultrasound visualization at 7 weeks of gestation of a gestational sac. Where a GnRH agonist was used for triggering final oocyte maturation, luteal support was associated with one oral tablet of 2 mg estradiol (Isdin, Novo Nordisk, Bagsvaerd, Denmark), 12/12 h for the same time of progesterone [24].

### Statistical analysis

Statistical analysis was carried out through the IBM SPSS Statistics 20 program for Windows. Means were compared by the *t*-Test for independent samples. Categorical variables were analysed using descriptive statistics and Chi-square test, with continuity correction. In some variables, in the presence of cells with expected < 5 value in contingency tables, the Fisher exact Test was used. Ratios were calculated using the Z-test for population proportions. All statistical tests were two-tailed, with significance level of 0.05 (p < 0.05).

## Results

Of the 4894 ART treatments analysed, there were 71/4894 (1.5 %) cycles with a suspection for development of OHSS and thus Buserelin was used for ovulation trigger, and all these cases have not developed OHSS. However, 51/4894 (1.0 %) cases evaded OHSS diagnosis and ovulation trigger was performed with HCG. These developed late OHSS, 28 (28/4894: 0.6 %; 28/51: 54.9 %) of moderate and 23 (23/4894: 0.5 %; 23/51: 45.1 %) of severe intensity, with the later requiring hospitalization. As all OHSS cases correspond to pregnant women they presented significant higher rates of implantation, pregnancy (biochemical, clinical and ongoing), live birth delivery (LBDR) and newborn (NB). Nevertheless the embryological and clinical results of these cases are presented for comparisons and in Tables.

Male and female ages (Table 1) were significant lower in the Buserelin and OHSS groups than the control group, with no differences between the Buserelin and OHSS groups. There was a significant lower time of infertility and a higher proportion of female factor in the Buserelin group in relation to the control group, with no differences between the control and OHSS groups or between the Buserelin and OHSS groups. Lower basal FSH (bFSH) levels were significantly lower in the Buserelin group regarding the control and OHSS groups, with no differences between control and OHSS groups. Cases with PCOS were significantly higher in the Buserelin group than in the control and OHSS groups, with the OHSS group being also significantly higher than the control group. Of the 51 OHSS cases, 15 had PCOS, 8 in the 28 cases with moderate OHSS (28.6 %) and 7 in the 23 cases with severe OHSS (30.4 %). The number of follicles was significantly higher in the Buserelin group than in control and OHSS groups, with the OHSS group being also significantly higher than the control group. As expected the total gonadotrophin dose was significantly lower in the Buserelin group regarding control and OHSS groups, with no differences between control and OHSS groups. The E2 serum levels were significantly higher in the Buserelin group regarding control and OHSS groups, being similar between the control and OHSS groups. No significant differences were observed regarding BMI or HCG dose (Table 1).

**Table 1:** Demographic and stimulation characteristics

Parameters	HCG	Buserelin	OHSS	p
*Cycles*	4772	71	51	
*Female age*	34.8 ± 4.5	32.3 ± 3.8	32.8 ± 3.5	a,b
*Male age*	36.6 ± 5.7	34.0 ± 4.1	34.1 ± 4.3	a,b
*Time infertility*	3.9 ± 3.1	3.1 ± 1.9	3.3 ± 2.2	a
*Male factor*	58.9	51.7	51.1	NS
*Female factor*	11.9	27.6	17.0	a
*Mixed factors*	29.2	20.7	31.9	a
*bFSH*	7.1 ± 3.1	5.7 ± 2.7	7.5 ± 3.7	a,c
*PCOS*	6.2	57.7	29.4	a,b,c
*BMI*	22.4 ± 3.7	22.1 ± 4.3	22.1 ± 4.4	NS
*Follicles*	6.1 ± 3.5	24.8 ± 8.8	18.1 ± 6.7	a,b,c
*Total dose*	2008.9 ± 986.3	1266. 1 ± 515.4	1716.2 ± 1000.6	a,c
*Time stimulation*	8.5 ± 1.7	8.3 ± 1.8	8.1 ± 1.3	NS
*Estradiol*	1238.3 ± 737.0	3565.9 ± 2592.9	1159.9 ± 711.9	a,c
*HCG dose*	9411.6 ± 1569.0	-	9482.8 ± 1549.7	NS
*Buserelin dose*	-	0.8 ± 0.0	-	

Values in “n”, rate or mean ± SD

Significance (p < 0.05)

a: HCG vs Buserelin

b: HCG vs OHSS

c: Buserelin vs OHSS

NS: not significant

HCG: group of patients without OHSS and oocyte trigger with HCG

Buserelin: group of patients without OHSS and oocyte trigger with Buserelin

OHSS: group of patients with OHSS and oocyte trigger with HCG

Female and male ages, Time of infertility, Male factor, Female factor and Mixed factors (years)

bFSH: basal FSH (mIU/ml)

PCOS: polycystic ovarian syndrome

BMI: body max index

Total dose: Total gonadotrophin dose (IU/ml)

Time of stimulation (days)

Estradiol at HCG day (pg/ml)

HCG dose (IU/ml)

Buserelin dose (IU/ml)

The mean number of retrieved oocytes (Table 2) was significantly higher in the Buserelin group than in control or OHSS groups, with the OHSS group being also significantly higher than the control group. However, the oocyte maturation rate was significantly lower in the Buserelin group, with no differences between the OHSS and control groups or between the Buserelin and OHSS groups. The fertilization rate was significantly higher in the OHSS group, with no differences with the Buserelin group. No significant differences were observed regarding the embryo cleavage rate and the mean number of embryos transferred. The rates of biochemical pregnancy and clinical pregnancy, and of implantation, were significantly higher in the OHSS group, with no significant differences between the Buserelin and control groups. No significant differences were found regarding the rates of twin pregnancies and triplet pregnancies, as well as in abortion. However, the Buserelin group evidenced a significant lower rate of singleton pregnancies in comparison with the control group, with no differences between the control and OHSS groups or between the Buserelin and OHSS groups. There was a significant higher rate of ectopic pregnancies in the Buserelin group regarding both OHSS and control groups, with no differences between the control and OHSS groups. The ongoing pregnancy rate was significantly higher in the OHSS group and significantly lower in the Buserelin group. The rates of delivery, LBDR and NB were significantly higher in the OHSS group regarding both control and Buserelin groups, with no differences between control and Buserelin groups. There were no significant differences in relation to the sex ratios. There was a significant higher rate of major NB malformations in the Buserelin group (two twins, each one with one NB with a major malformation: Kabuki syndrome and cardiac hypoplasia with surgery), with no cases of NB malformations in the OHSS group. The Buserelin and OHSS groups did not present any case of stillbirths, NB chromosome abnormalities or early neonatal death (Table 2).

**Table 2:** Embryological and clinical outcomes

Parameters	HCG	Buserelin	OHSS	p
*Cycles*	4772	71	51	
*COC*	36768	1134	563	
*COC (mean)*	7.8 ± 4.7	16.0 ± 6.9	11.0 ± 4.0	a,b,c
*MII*	30872	910	469	
*Maturation rate (MII/COC)*	84.0	80.2	83.3	a
*2PN/2 PB*	21410	648	346	
*Fertilization rate (2PN/MII)*	69.4	71.2	73.8	b
*Embryos cleaved*	20954	641	337	
*Embryo cleavage rate (d2/2PN)*	97.9	98.9	97.4	NS
*Embryo transfer cycles (ETC.)*	4391	67	51	
*n° Embryos transferred (mean)*	8443-1.9 ± 0.5	121-1.8 ± 0.4	92-1.8 ± 0.4	NS
*Biochemical pregnancy (/ETC.)*	1973-44.9	32-47.8	49-96.1	b,c
*Clinical pregnancy (CP) (/ETC.)*	1709-38.9	20-29.9	48-94.1	b,c
*Sacs*	2155	23	64	
*Implantation rate (sacs/n°ET)*	25.5	19.0	69.6	b,c
*Singletons (/CP)*	1197-70.0	9-45.0	29-60.4	a
*Twins (/CP)*	473-27.7	7-35.0	16-33.3	NS
*Triplets (/CP)*	11-0.6	0	1-2.1	NS
*Ectopic pregnancy (/CP)*	28-1.6	4-20.0	2-4.2	a,c
*Abortion (/CP)*	253-14.8	2-10.0	6-12.5	NS
*Ongoing pregnancy (/ETC.)*	1428-32.5	14-20.9	40-78.4	a,b,c
*Delivery (/ETC.)*	1329-30.3	14-20.9	40-78.4	b,c
*Stillbirths (/ETC.)*	1-0.02	0	0	NS
*Live-birth delivery rate (/ETC.)*	1328-30.2	14-20.9	40-78.4	b,c
*Newborn (NB) (/ETC.)*	1655-37.7	19-28.4	53-100	b,c
*Male (/NB)*	832-50.5	11-57.9	25-47.2	NS
*Female (/NB)*	817-49.5	8-42.1	28-52.8	NS
*Male/Female ratio*	1.0	1.4	0.90	NS
*Newborn malformations (/NB)*	36-2.2	2-10.5	0	a,c
*Major (/NB)*	27-1.6	2-10.5	0	a,c
*Minor (/NB)*	9-0.5	0	0	NS
*NB Chromosome abnormalities (/NB)*	5-0.3	0	0	NS
*Early neonatal death (/ETC.)*	4-0.1	0	0	NS

Values in “n”, rate or mean ± SD

Significance (p < 0.05)

a: HCG vs Buserelin

b: HCG vs OHSS

c: Buserelin vs OHSS

NS: not significant

HCG: group of patients without OHSS and oocyte trigger with HCG

Buserelin: group of patients without OHSS and oocyte trigger with Buserelin

OHSS: group of patients with OHSS and oocyte trigger with HCG

COC: cumulus-oocyte complexes (aspirated oocytes)

MII: mature oocytes at metaphase II of meiosis

2PN/2 PB: normal fertilized oocytes (with 2 pronuclei and 2 polar bodies)

There were no significant differences concerning the gestational age and NB weight (Table 3). There was 1 case of extremely preterm in the OHSS group, without any cases of very preterm in the Buserelin group. In both the Buserelin and OHSS groups there were no cases of very low weight (Table 3).

**Table 3:** Newborn outcomes

Parameters	HCG	Buserelin	OHSS	p
*Cycles*	4772	71	51	
*ETC.*	4391	67	51	
*Newborn*	1655-37.7	1928.4	53-100	b,c
*Gestation age (weeks)*	37.4 ± 2.7	38.1 ± 2.4	37.3 ± 2.7	NS
*Term*	38.5 ± 1.1	39.3 ± 1.6	38.4 ± 1.2	a
*Preterm (PT)*	33.7 ± 3.0	35.1 ± 1.3	33.3 ± 2.6	NS
*Very PT*	28.7 ± 2.5	(0)	27.0 (1)	a
*Extremely PT*	25.1 ± 1.7	(0)	27.0 (1)	a
*Weight (g)*	2709.0 ± 705.6	2717.4 ± 693.0	2529.6 ± 726.4	NS
*Normal weight*	3086.1 ± 352.2	3127.3 ± 570.9	3078.6 ± 379.7	NS
*Low weight (LW)*	1900.4 ± 507.3	2073.3 ± 160.1	1914.8 ± 485.6	NS
*Very LW*	1048.6 ± 280.5	(0)	1193.7 ± 261.5	a,c
*Extremely LW*	756.0 ± 193.2	(0)	861.0 ± 66.5 (2)	a

Values in “n” and mean ± SD

Significance (p < 0.05)

a: HCG vs Buserelin

b: HCG vs OHSS

c: Buserelin vs OHSS

NS: not significant

(0), (1) and (2): number of cases

HCG: group of patients without OHSS and oocyte trigger with HCG

Buserelin: group of patients without OHSS and oocyte trigger with Buserelin

OHSS: group of patients with OHSS and oocyte trigger with HCG

With regard to the 23 severe OHSS cases, the average hospital stay was of 8.2 days (2–15). All patients presented at entry bloating and abdominal discomfort, nausea, fatigue and dyspnea. Blood tests showed hemoconcentration and ultrasound revealed ovarian enlargement (right ovary: 7.89 cm, 5.5-10 cm; Left ovary: 6.9 cm, 6.3-8.2 cm), with moderate to massive ascites. This made a diagnosis of late (after positive βHCG) and severe OHSS. Patients received fluid therapy (to lower hemoconcentration), furosemide (to force diuresis, as fluid therapy increases the risk of ascites and pleural effusion and OHSS is associated with increased vascular permeability; also, an increased intra-abdominal pressure can also compress kidneys and impair renal function), and enoxaparin (due to risk of thromboembolism derived from hemoconcentration). The bladder was probed with flowmeter for registration of diuresis (most women had some difficulty in urinating on entry), and a record was made up of the hydroelectrolytic balance. During hospitalization, patients had no further complications, and paracentesis or culdocentese were not necessary. Patients were discharged with indication for rest, fluid restriction, proper diet, paracetamol (SOS), and immediate referral if they had any signs or symptoms of worsening situation, which did not occur.

## Discussion

Ovarian hyperstimulation syndrome has been reported to occur in about 0.2-2.6 % of ART treatment cycles [4, 25]. Previous reports evidenced 0.7 % of moderate and 0.3 % of severe OHSS [26], whereas others reported 2.1 % of severe OHSS, with 1.2 % of early and 0.9 % of late onset [10]. Our results are in accordance with these later values as of 4894 ART treatment cycles we had 1.5 % of cases with suspected OHSS that was efficiently avoided with the use of a GnRH agonist for ovulation trigger, and 1 % of cases with late onset OHSS, of which 0.6 % were of moderate and 0.5 % of severe intensity.

Lower female age was associated with OHSS [1, 2, 10, 11, 27] and suggested to be related with a larger number of recruitable follicles and a higher density of gonadotrophin receptors, thus enabling a more marked response to the same. We also found that a lower female age was associated with OHSS and this might be related with the observed significant lower bFSH levels in cases with risk for developing OHSS, although another report did not find any significant difference regarding bFSH [28]. However, female age is considered unsuitable as predictive factor since no cut-off value was still defined [4, 16, 28]. Although not previously referred, we observed a similar association of OHSS with a lower male age and time of infertility, which might be associated with the younger age of the couples.

We observed a significant higher number of female factor cases in association with the risk of OHSS development that might be associated with the increased rate of PCOS cases (58 %). Although infertility causes have not been related to OHSS [10], PCOS is one factor clearly associated with predisposition for developing OHSS. The predisposition of PCOS patients to OHSS is probably due to the fact that the higher antral follicle pool has an increased sensitivity to gonadotropin stimulation [1, 2, 4, 10, 11, 27, 29].

Contrary to previous reports that found an association between decreased BMI and development of OHSS [1, 2, 11, 16, 27], we and other authors [4, 10, 28, 30] did not find a significant difference.

The presence of a large number of developing follicles has been considered a risk factor for OHSS [1, 10, 11, 27]. However, due to the discrepancies in different studies, even this parameter is per-se not considered convincing [4]. A fundamental work suggested a solution to this problem indicating two cut-offs, one when there were ≥ 13 follicles with ≥ 11 mm, which presented 85.5 % of sensitivity and 69 % of specificity, and the other for E2 concentration levels > 2560 pg/ml, which presented 53 % of sensitivity and 77 % of specificity. In cases where controlled ovarian hyperstimulation is performed with a GnRH antagonist these values were incremented to > 18 follicles and E2 concentration levels > 5000 pg/ml, with 83 % of sensitivity and 84 % of specificity [10]. In the present work, ovarian stimulation was performed with a GnRH antagonist and in all cases where Buserelin was used for ovulation trigger the mean number of follicles was 24.8 ± 8.8 and the mean E2 concentration levels was 3565.9 + 2592.9. However, in our cases that developed late onset OHSS after HCG being used for ovulation induction the mean number of follicles was above (18.1 ± 6.7) the proposed cut-off for follicles, but the mean E2 levels were below (1159.9 ± 711.9) the suggested cut-off.

The number of collected oocytes has also been suggested to be related with OHSS, but the suggested cut-off of ≥ 20 oocytes is disputable [4, 10]. Our present results showed that OHSS can develop even with a mean number of collected oocytes of 11.0 ± 4.0, although those cases with suspected risk for OHSS presented a mean number of 16.0 ± 6.9.

Regarding the total gonadotrophin dose, the time of stimulation and the HCG dose, we did not observe any relationship to OHSS, which is in accordance with previous observations [10, 28]. The young age of the ovaries with predisposition for a better response to stimulation has been implicated to explain the high levels of E2 observed, with E2 having been considered the best defined predictor for OHSS [1, 2, 10, 11, 27, 28]. However, with the use of a GnRH antagonist for ovarian stimulation, E2 concentrations became less reliable for OHSS prediction [4, 10]. In our ovarian stimulation we used the antagonist protocol and previous reports have demonstrated that this protocol results in a lower number of OHSS cases [31], which reassures our stimulation strategy. A possible explanation for these findings is that the antagonist largely reduces the duration of the GnRH analogue and prevents events related to the flare-up or down-regulation induced by agonists. It also requires less amounts of FSH, and the E2 production per follicle and the E2 concentration before HCG administration is also lower [32]. Thus, although mean E2 concentrations are significantly higher in patients who develop OHSS, the maximum value alone is not a sufficient accurate predictive factor [4, 10]. Our results thus suggest the same, as our 51 cases that developed OHSS had similar mean levels of E2 concentrations (1159.9 ± 711.9 pg/ml) to those observed in our control group (1238.3 ± 737.0 pg/ml), which means that the suggested E2 levels above 3500–6000 pg/ml to be predictive for OHSS development [4, 10] are not sufficiently accurate.

Taking all these factors into consideration, it was suggested that the best way to predict development of OHSS was to combine several factors, such as: E2 concentration levels > 4500 pg/ml), number of follicles ≥ 13 with ≥ 11 mm, and oocytes collected > 15). With the use of a GnRH antagonist for controlled ovarian hyperstimulation these values were suggested to raise to E2 concentration levels of > 5000 pg/ml and to a number of follicles > 18. However, even with these cut-off values it has not been possible to totally avoid development of OHSS [4, 10, 11]. Our results suggest the same, as in our 51 cases that developed OHSS, the mean E2 concentrations were below the referred < 1200 pg/ml, the mean number of follicles was 18.1 ± 6.7 and the mean number of retrieved oocytes was 11.0 ± 4.0.

The use of an agonist instead of HCG for ovulation induction can lead to elimination of OHSS [13–15, 24]. However, as it causes corpus luteum deficiency and a defective luteal phase of the endometrium (reduced endogenous progesterone production and low LH secretion around the time of implantation), the luteal phase is supplemented with oral E2 besides vaginal progesterone. Later, it was suggested further supplementation with a bolus of low dose HCG at oocyte pick-up [13, 28]. As LH is responsible for the steroidogenic activity of the corpus luteum, up-regulation of several factors involved in implantation and activation of endometrial LH receptors, this HCG bolus finally rescued the luteal phase [13, 28]. As an alternative, after avoiding OHSS with the use of an agonist trigger, the fresh embryo transfer is canceled and all oocytes or embryos are frozen. In a later cycle, with a suited prepared endometrium or in a natural cycle, embryos are thawed and transferred [15]. Although two cases of severe OHSS have been described after agonist trigger, the authors speculated whether GnRH receptor, FSH receptor or LH receptor gene mutations have led to and OHSS predisposition [33].

Our results refer to a period where the use of a GnRH agonist for ovulation trigger was still performed without a bolus of HCG at ovum pick-up for luteal supplementation. Nevertheless, we believe that it would be important to report that in the absence of that type of supplementation the embryological, clinical and NB outcomes were not compromised.

There is only one previous study evaluating similar outcomes, although without providing all parameters that we present in this report. In that study, 152 patients were treated with a GnRH agonist for ovulation trigger, followed by a HCG bolus at oocyte pick-up for luteal rescue, and compared with 150 patients treated with HCG for ovulation trigger. Comparisons between the two groups did not show any significant differences towards the rates of oocyte maturation, fertilization, biochemical pregnancy, clinical pregnancy, ongoing pregnancy and delivery, with 2 % of OHSS cases occurring in the HCG group [28].

Our present results thus represent the first study that gives full demographic, stimulation, embryological, clinical and NB outcomes comparing ovulation trigger with HCG vs with Buserelin in cases with suspicious OHSS without the use of a HCG bolus for luteal rescue. With the use of a GnRH agonist as a ovulation trigger we obtained lower rates of oocyte maturation (84 % vs 80 %) and ongoing pregnancy (33 % vs 21 %), but we attained similar rates regarding fertilization (69 % vs 71.2 %), embryo cleavage (98 % vs 99 %), biochemical (45 % vs 48 %) and clinical ((39 % vs 30 %) pregnancies, implantation (26 % vs 19 %), abortion (15 % vs 10 %), live birth delivery (30 % vs 21 %) and NB (38 % vs 28 %) rates, and also with no interference with the gestational age and weight of NB. Furthermore, our rate of OHSS development was 1 %.

However, the use of the GnRH agonist was associated with significant higher rates of ectopic pregnancy and major NB malformations. Although our cases treated with the GnRH agonist did not have any case of OHSS, the results were probably due to the insufficient quality of the endometrium (lutein insufficiency with an abnormal endometrium out of the implantation phase) as we did not use the HCG bolus at ovum pick-up for luteal supplementation. This may also explain the higher rate of ectopic pregnancy, as the embryo did not find the appropriate endometrial milieu. Regarding the higher rate of NB malformations (two cases) this cannot be attributed to the GnRH agonist as that was not observed in the long protocol that uses a GnRH agonist for stimulation. Thus, this rate has to be attributed to the low number of cases analysed.

## Conclusions

We found that young age, lower time of infertility, lower bFSH, higher female factor and PCOS, and high number of follicles and of E2 concentration were the risk factors found associated with OHSS. The elevated serum E2 levels and the number of follicles were used to predict development of OHSS and in 71 cases Buserelin was used effectively, with no cases of OHSS. Nevertheless 51 cases evaded this diagnosis and developed late onset OHSS, with 23 needing hospitalization, although with no major complications. These later cases also presented higher follicle count but the E2 levels were within the normal range. It thus remains to develop more strict criteria to avoid all cases with OHSS as previously suggested [4, 10–13, 15, 24, 26, 28, 34].
